# Comparing health insurance data and health interview survey data for ascertaining chronic disease prevalence in Belgium

**DOI:** 10.1186/s13690-020-00500-4

**Published:** 2020-11-17

**Authors:** Finaba Berete, Stefaan Demarest, Rana Charafeddine, Olivier Bruyère, Johan Van der Heyden

**Affiliations:** 1 SD Epidemiology and public health, Sciensano, Juliette Wytsmanstraat, 14 1050 Brussels, Belgium; 2 Department of Public Health, Epidemiology and Health Economics, University of Liège, Liège, Belgium; 3 WHO Collaborating Centre for Public Health aspects of musculoskeletal health and ageing, Department of Public Health, Epidemiology and Health Economics, University of Liege, Liège, Belgium

**Keywords:** Chronic diseases, Health administrative data, Data linkage, Validity, HEALTH insurance data, Chronic diseases ascertainment

## Abstract

**Background:**

Health administrative data were increasingly used for chronic diseases (CDs) surveillance purposes. This cross sectional study explored the agreement between Belgian compulsory health insurance (BCHI) data and Belgian health interview survey (BHIS) data for asserting CDs.

**Methods:**

Individual BHIS 2013 data were linked with BCHI data using the unique national register number. The study population included all participants of the BHIS 2013 aged 15 years and older. Linkage was possible for 93% of BHIS-participants, resulting in a study sample of 8474 individuals. For seven CDs disease status was available both through self-reported information from the BHIS and algorithms based on ATC-codes of disease-specific medication, developed on demand of the National Institute for Health and Disability Insurance (NIHDI). CD prevalence rates from both data sources were compared. Agreement was measured using sensitivity, specificity, positive predictive value (PPV) and negative predictive value (NPV) assuming BHIS data as gold standard. Kappa statistic was also calculated. Participants’ sociodemographic and health status characteristics associated with agreement were tested using logistic regression for each CD.

**Results:**

Prevalence from BCHI data was significantly higher for CVDs but significantly lower for COPD and asthma. No significant difference was found between the two data sources for the remaining CDs. Sensitivity was 83% for CVDs, 78% for diabetes and ranged from 27 to 67% for the other CDs. Specificity was excellent for all CDs (above 98%) except for CVDs. The highest PPV was found for Parkinson’s disease (83%) and ranged from 41 to 75% for the remaining CDs. Irrespective of the CDs, the NPV was excellent. Kappa statistic was good for diabetes, CVDs, Parkinson’s disease and thyroid disorders, moderate for epilepsy and fair for COPD and asthma. Agreement between BHIS and BCHI data is affected by individual sociodemographic characteristics and health status, although these effects varied across CDs.

**Conclusions:**

NHIDI’s CDs case definitions are an acceptable alternative to identify cases of diabetes, CVDs, Parkinson’s disease and thyroid disorders but yield in a significant underestimated number of patients suffering from asthma and COPD. Further research is needed to refine the definitions of CDs from administrative data.

**Supplementary Information:**

The online version contains supplementary material available at 10.1186/s13690-020-00500-4.

## Background

Chronic diseases (CDs) represent an important concern for public health policy. Indeed, their prevalence is constantly increasing and they are by far the leading cause of mortality in Europe, representing 77% of the total disease burden and 86% of all deaths [1].

An important prerequisite for the CDs management is to be able to identify, in a valid, simple and inexpensive way, the population with CDs that need proactive and planned care [2]. For this purpose, population-based data for routine monitoring of CDs prevalence are fundamental to describe the burden of disease and to plan and evaluate disease prevention, treatment and management strategies and by defining target populations [3, 4].

Prevalence of CDs is often estimated using population health surveys, disease registers, hospitalization or outpatient records [3–8]. Besides these traditional methods, health administrative databases have been used as an alternative, efficient source of data for CDs surveillance [4, 5, 9, 10]. Health administrative databases can be accessed easily and quickly, associated costs are low and they are quite exhaustive. In some cases such databases can be used to provide cross-sectional and longitudinal data on the prevalence and incidence of diseases in the entire population [10]. The use of such data is very challenging [11] yet due to the opportunity they provide, they have often been used for surveillance purposes. For instance, in France, the French national health insurance information system (Système National de Données de Santé – SNDS) has been used to develop the Diabetes National Surveillance System which serves as a base to estimate the national prevalence of pharmacologically treated diabetes and the incidence of diabetes-related complications, as well as their temporal trends and their territorial variations [12]. To estimate these indicators, a diabetes case definition algorithm based on antidiabetic drug consumption was applied [4]. Drug use data, especially prescription drugs, have also been frequently used to estimate CDs prevalence [5, 7, 13].

In Belgium, the prevalence of specific CDs is usually assessed, based on data gathered through the Belgian health interview survey (BHIS), conducted every 5-years. Next to this, other sources such as hospital discharge data, disease-specific registries (e.g., Belgian cancer registry), sentinel practice networks (e.g., Intego sentinel GP network), also represent important tools to obtain prevalence/incidence rates of CDs.

More than 99% of the Belgian population is covered by the Belgian compulsory health insurance (BCHI). The BCHI database provides detailed and complete information on the reimbursement of health care costs for almost the entire population. Such information is widely used by important actors in the health field, such as the National Institute for Health and Disability Insurance (NIHDI), the Belgian health care knowledge centre and the Federal planning bureau tor studying and planning topics mainly related to health care costs and expenditures. Although these data are not meant for epidemiological purposes, BCHI data are also used to estimate the prevalence of some CDs at population level [14].

At the initiative of the NIHDI, a panel of experts (mainly clinicians) have developed algorithms based on prescribed medication dispensed in public pharmacies to construct indicators of CDs. The algorithms are all based on a minimum consumption of 90 DDD (Defined Daily Dose) during one calendar year of drugs of certain (sub) classes of ATC (Anatomical Therapeutic Chemical), often in combination with the minimum age of the patient [15].

These indicators of CDs are useful for the NIHDI, to identify specific patient populations. However, since their development, they have only been validated qualitatively. To our knowledge, only one study has compared the prevalence of diabetes mellitus and thyroid disorders from BHIS, BCHI and diagnostic codes in Flanders [6].

The main objective of this study was to assess agreement between health administrative and self-reported cases definitions of diabetes, asthma, chronic obstructive pulmonary disease (COPD), cardiovascular diseases including hypertension (CVDs), Parkinson’s disease, thyroid disorders and epilepsy in the Belgian population, assuming self-reported data as a gold standard. The aforementioned CDs were chosen because they are common diseases with a lower risk of misreporting by BHIS participants and because they are generally treated with specific drugs which are more or less specific for the disease. Furthermore, we also sought to determine the subject sociodemographic and health status characteristics that may affect the agreement between the two data sources.

## Methods

### Study design and population

This is a descriptive cross-sectional study. The study population included all participants of the Belgian health interview survey (BHIS) 2013 aged 15 years and older (*n* = 9112).

### Data sources

Date were derived from the HISLINK 2013 study, an individual linkage between the Belgian health interview survey (BHIS) 2013 data and the Belgian compulsory health insurance data (BCHI) from 2012 to 2018.

The BHIS is a national, cross-sectional household survey conducted every 5 years since 1997 by Sciensano, the Belgian health institute, among a representative sample of Belgian residents. Participants are selected from the national population register through a multistage stratified sampling procedure. The participation rate in the survey was 57% at the household level. In the BHIS, information is collected on health status, health behavior, health care consumption, sociodemographic characteristics and use of medicines. The detailed methodology of the survey is described elsewhere [16].

The BCHI data contain exhaustive and detailed information on the reimbursed health expenses of over 99% of the total population. The database also includes a limited amount of socio-demographic information. The BCHI data were provided by the Intermutualistic Agency (IMA). IMA is a joint venture of the seven national sickness funds and collects and manages all data on healthcare expenditures as well as prescription information on reimbursed medicines (Pharmanet data) [17]. Pharmanet logs all data on reimbursed dispensed medication from public pharmacies in Belgium. Pharmanet data include information on the date of dispensing, the quantity per package (QPP), the daily defined dose (DDD) and the national code number of the medicine (CNK codes) which allows to link each medicine to its ATC-code. The list of ATC codes per CNK codes was provided by the NIHDI.

Individual BHIS 2013 data were linked with BCHI data using the unique national register number. The study population included all participants of the BHIS 2013 aged 15 years and older (*n* = 9112). The linkage was possible for 93% of them, resulting in a final sample of 8474 individuals. The HISLINK 2013 was used because it was the most recent linked database available at the moment of this study.

### Identification of chronic diseases

The prevalence information from BHIS was collected using a list of CDs (35 in total) based on the following question: *“Have you suffered during the last 12 months from the following disease?”.* Since there is no specific indicator for CVDs in the BHIS, we considered a person to have CVDs (including hypertension) when they reported having had in the past 12 months at least one of the following CDs: myocardial infarction, coronary disease, hypertension, stroke, or other serious heart diseases.

In the BCHI data, the NIHDI algorithms were used to ascertained cases of CDs. In these algorithms, CDs cases were identified based on the ATC-codes of dispensed medication in public pharmacies, using the WHO guidelines on the ATC classification system [18]. So, a CD was assigned to a participant if the total of DDDs reimbursed for all selected ATC-codes used in the treatment for this CD is greater or equal to 90 [15] in the past 12 months preceding the participation in the BHIS. The selected ATC-codes for each CD are presented in Table 1.

**Table 1:** Survey questions and ATC prescription drug codes for chronic disease case ascertainment, HISLINK 2013, Belgium

Chronic diseases	Survey questions: “Have you suffered during the last 12 months from … “	ATC-codes
Diabetes mellitus	Diabetes?	A10A A10B
Cardiovascular diseases	Myocardial infarction?	C01
	Coronary disease?	C02
	Hypertension?	C03
	Stroke?	C07
	Other serious heart disease?	C08 C09
COPD	COPD?	R03BB R03DA04 R03A^a^ R03BA^a^
Asthma	Asthma?	R03DC01 R03DC03 R03DX05 R03A^b^ R03BA^b^
Parkinson’s disease	Parkinson’s disease?	N04AB N04AC N04B
Epilepsy	epilepsy?	N03
Thyroid disorders	thyroid disorders?	H03AA

^a^ For people aged < = 50 years

^b^ For people aged > 50 years

### Statistical analyses

We calculated the weighted prevalence rates from both data sources for the 7 selected CDs. The delta method [19] was applied to test if there was a significant difference between the estimates of both sources.

The agreement was measured by estimating sensitivity, specificity, positive predictive value (PPV) and negative predictive value (NPV) and their 95% CI, assuming BHIS data as gold standard. Sensitivity was defined as the percentage of true positive cases an algorithm detects among all positive disease cases. Positive disease cases are BHIS respondents who reported having the specified disease. Specificity was defined as the percentage of true negative cases an algorithm detects among all the negative disease cases. Negative disease cases are BHIS respondents who did not report having the specified disease. Positive predictive values (PPVs) and negative predictive values (NPVs) are also reported for each chronic disease algorithm. PPV refers to the percentage of individuals with a positive result for an algorithm among those who reported having the disease. NPV refers to the percentage of individuals with a negative result for an algorithm who did not report having the disease [20].

Furthermore, Kappa values were calculated to differentiate between true agreement and agreement produced by chance. Kappa values were interpreted as follows: κ ≤ 0.40, fair-to-poor agreement; κ = 0.41 to 0.60, moderate agreement; κ = 0.61 to 0.80, substantial agreement; and κ = 0.81 to 1.00, almost perfect agreement [21].

Sensitivity analyses were conducted by repeated analyses for different cut-off points of the DDD for all the CDs.

Finally, univariable and multivariable logistic regression analysis were performed for each CD (except for the Parkinson’s disease because of small number of cases unable to provide reliable estimates) to further investigate the effect of respondent’s characteristics on the total agreement (true positive or true negative) between BHIS and BCHI data sources. Participants characteristics included in the model are: gender, age-group (15–34, 35–54, 55–74 and 75+ years), education (low, intermediate, high), nationality (Belgian, EU-countries, other countries), household income (quintile), region of residence (Flanders, Brussels, Wallonia), self-perceived health (good to very good, very bad to fair), presence of multimorbidity (yes/no) and polypharmacy defined as simultaneous use 5 medicines or more on a typical day (yes/no).

A two-sided alpha level of 0.05 was considered statistically significant. All analyses were performed using SAS 9.4 (SAS Institute Inc., Cary, NC, USA) and Stata 16.1 and taking into account the survey settings.

### Ethics statement

As mentioned above, this study was carried out using the individual linkage between the BHIS 2013 data and the BCHI data. The BHIS 2013 was carried out in line with the Belgian privacy legislation and has been approved by the ethics committee of the University hospital of Ghent on October, 1st 2012 (advice EC UZG 2012/658). The participation to BHIS is voluntary. There was no formal written and signed consent foreseen as participation was considered as consent. In addition, for the data linkage, an authorization was obtained from the Information Security Committee (local reference: Deliberation No. 17/119 of December 19, 2017, amended on September 3, 2019).

This study is reported according to the STROBE statement.

## Results

Table 1 summarizes the CDs with identification questions in the BHIS data source and the assigned ATC-codes in the BCHI data source.

Characteristics of the study population, unweighted and weighted to reflect the general Belgian population in terms of age, gender and region are presented in Table A1 (supplementary material). More than half of the population perceived their health to be good to very good, 15% suffers from multimorbidity and one person out of ten simultaneous uses 5 medicines or more on a one day reference period.

Table 2 shows the prevalence of CDs in the population by data source. The prevalence rates obtained from administrative data source were significantly higher than those obtained from survey data for CVDs (including hypertension), but on the contrary, they were significantly lower for COPD and asthma. No significant difference was found between the two data sources for the remaining CDs.

**Table 2:** Prevalence (weighted percentages) of chronic diseases among the population aged 15 years and over by data source, HISLINK 2013, Belgium

Chronic disease	Prevalence in BHIS (E1)	Prevalence in BCHI (E2)	Absolute difference^b^ (E1-E2)	Relative difference^b^ (E1-E2)/E2
	% (95% CI)	% (95% CI)	% (95% CI)	% (95% CI)
Diabetes mellitus	5.46 (4.78 to 6.15)	5.69 (5.05 to 6.33)	−2.25 (−1.13 to 6.84)	−3.96 (− 19.65 to 11.73)
CVDs^*a*^	19.15 (17.88 to 20.42)	25.09 (23.68 to 26.51)	−5.94 (−7.68 to −4.20)	− 23.68 (− 29.79 to − 17.57)
COPD^*a*^	4.01 (3.45 to 4.56)	2.82 (2.35 to 3.29)	1.19 (0.47 to 1.90)	42.10 (11.85 to 72.35)
Asthma^*a*^	4.36 (3.77 to 4.96)	1.64 (1.29 to 1.99)	2.72 (2.05 to 3.39)	165.82 (99.15 to 232.49)
Parkinson’s disease	0.50 (0.28 to 0.71)	0.38 (0.21 to 0.55)	0.11 (− 0.16 to 0.39)	29.77 (− 50.95 to 110.49)
Epilepsy	0.94 (0.64 to1.24)	1.33 (1.03 to 1.68)	−0.38 (− 0.80 to 0.03)	−28.98 (− 55.85 to 2.13)
Thyroid disorders	5.89 (5.20 to 6.58)	5.43 (4.78 to 6.08)	0.46 (−0.49 to 1.42)	8.57 (−97.72 to 26.91)

^a^Denotes significant difference between BHIS prevalence en BCHI prevalence

^b^Computed before rounded the estimated prevalences

*CVDs* cardiovascular diseases (including hypertension), *COPD* chronic obstructive pulmonary disease

The agreement measures are described in Table 3. Sensitivity was good for CVDs (83%), fair for diabetes (78%) and poor for all other CDs (value varying between 27 and 67%). The specificity was excellent for all CDs (specificity above 98%) except for CVDs (specificity = 89%). The PPV was poor to fair for all the CDs (PPV varying between 41 and 75%), except for Parkinson’s disease where it was good (PPV = 83%). Irrespective of the CDs, the NPV was excellent (NPV varying between 96 and 99%). The Kappa statistic was good for diabetes, CVDs, Parkinson’s disease and thyroid disorders (kappa between 0.63 and 0.77), moderate for epilepsy (kappa = 0.46) and fair for COPD and asthma (kappa = 0.35).

**Table 3:** Agreement between self-reported chronic disease and definitions from administrative data, HISLINK 2013, Belgium*

Chronic disease	Sensitivity (%) (95% IC)	Specificity (%) (95% IC)	PPV (%) (95% IC)	NPV (%) (95% IC)	Kappa (95% CI)
Diabetes mellitus	78.5 (72.1–85.0)	98.5 (98.2–98.9)	75.4 (70.5–80.3)	98.8 (98.3–99.2)	0.77 (0.75–0.80)
CVDs	83.1 (80.6–85.6)	88.6 (87.6–89.7)	63.4 (60.6–66.2)	95.7 (95.0–96.3)	0.63 (0.61–0.65)
COPD	28.8 (22.3–35.3)	98.3 (97.9–98.6)	40.9 (32.5–49.3)	97.1 (96.6–97.5)	0.35 (0.30–0.40)
Asthma	27.4 (21.3–33.6)	99.5 (99.3–99.7)	72.9 (63.8–82.1)	96.8 (96.2–97.3)	0.35 (0.30–0.41)
Parkinson’s disease	64.3 (38.9–89.8)	99.9 (99.9–100)	83.5 (69.8–97.3)	99.8 (99.7–100)	0.70 (0.58–0.82)
Epilepsy	60.4 (44.6–76.2)	99.2 (99.0–99.4)	42.9 (31.0–54.8)	99.6 (99.4–99.8)	0.46 (0.37–0.55)
Thyroid disorders	66.7 (61.0–72.4)	98.4 (98.1–98.7)	72.4 (67.3–77.5)	97.9 (97.5–98.3)	0.66 (0.62–0.69)

*CVDs* cardiovascular diseases (including hypertension), *COPD* chronic obstructive pulmonary disease, *PPV* positive predictive value, *NPV* negative predictive value

* Sensitivity, specificity, PPV and NPV presented with self-reported as the referent

**Fig. 1:**
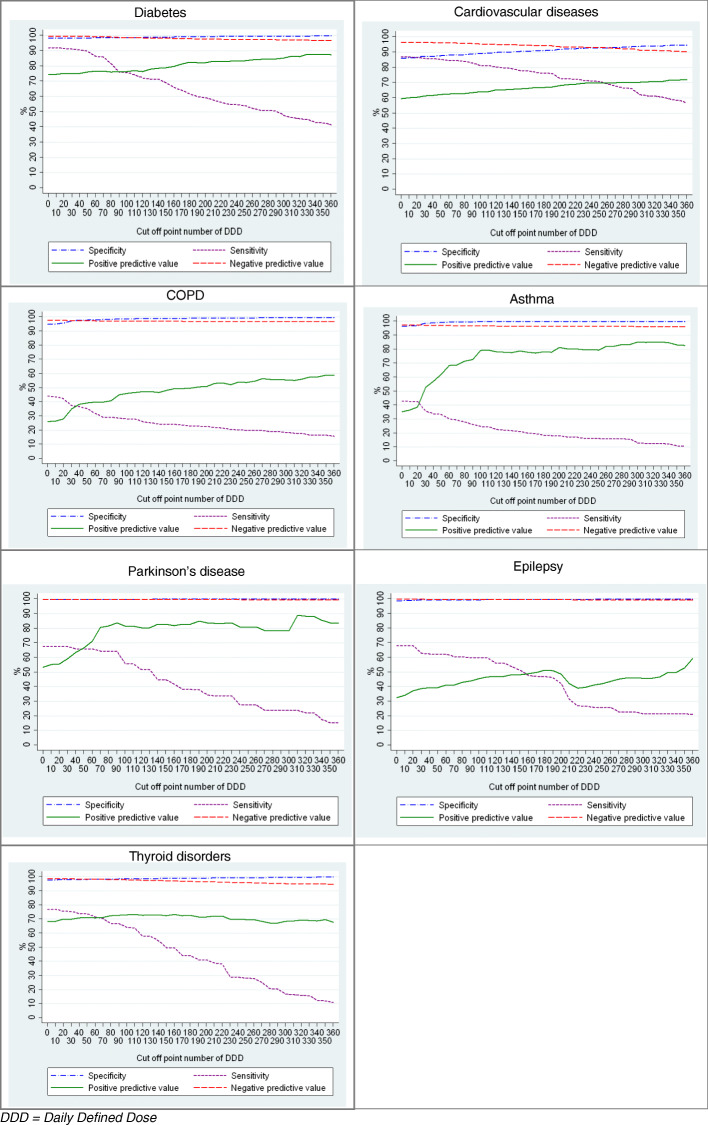
Sensitivity analysis: validity measures of chronic diseases as a function of the DDD threshold, HISLINK 2013. DDD = Daily Defined Dose

The results of the sensitivity analysis are presented in Fig. 1. Across the CDs, the sensitivity decreased with the increase of the cut-off point of the DDD, while the PPV slightly increased after the threshold of 90 DDD. Notable for Parkinson’s disease was the highest PPV around 320 DDDs and for thyroid disorders was the lowest PPV around 220 DDDs.

Table 4 shows the results from the multivariable logistic regression, while the unadjusted odds ratios are presented in additional Table A2 (supplementary material). Table 4 shows that the agreement between BHIS and BCHI data sources is affected by individual sociodemographic characteristics and health status. However, the characteristics which are associated, the magnitude and direction of the effect varied across CDs. For instance, gender was not significantly associated with the agreement between BHIS and BCHI data except for thyroid disorders where the agreement was significantly lower among women (OR: 0.26, 95% CI: 0.17–0.40). Compared to the reference age-group (55–74 years), belonging to the youngest age-group (15–34 years) was associated with a greater level of agreement between the data sources for diabetes (OR: 6.40, 95% CI: 2.38–17.25), CVDs (OR: 8.63, 95% CI: 5.56–13.39) and thyroid disorders (OR: 2.76, 95% CI: 1.54–4.95), while the reverse is true for asthma (OR: 0.19, 95% CI: 0.10–0.36). Regarding participant’s health status, people with a relatively good subjective health, those without multimorbidity and those who didn’t simultaneous use 5 medicines or more on a typical day (polypharmacy) have greater odds of agreement between the two sources except for CVDs where the absence of multimorbidity was significantly associated with a lower odds of agreement.

**Table 4:** Odds Ratios* (95% CIs) for predictors of agreement between administrative and survey data for chronic diseases, HISLINK 2013, Belgium

	Diabetes	CVDs	COPD	Asthma	Thyroid disorders
**Gender**					
Male	Ref.	Ref.	Ref.	Ref.	Ref.
Female	1.21 (0.71–2.06)	0.92 (0.73–1.15)	0.89 (0.62–1.27)	1.28 (0.88–1.86)	0.26 (0.17–0.40)^a^
**Age group**					
15–34	6.40 (2.38–17.25)^a^	8.63 (5.56–13.39)^a^	1.19 (0.55–2.56)	0.19 (0.10–0.36)^a^	2.76 (1.54–4.95)^a^
35–54	1.09 (0.56–2.10)	2.02 (1.50–2.72)^a^	0.81 (0.51–1.26)	0.51 (0.30–0.87)^a^	1.59 (0.98–2.58)
55–74	Ref.	Ref.	Ref.	Ref.	Ref.
75+	1.16 (0.54–2.47)	0.47 (0.35–0.63)^a^	0.98 (0.61–1.55)	2.09 (1.13–3.84)^a^	1.01 (0.60–1.69)
**Education**					
Low	Ref.	Ref.	Ref.	Ref.	Ref.
Intermediate	0.81 (0.42–1.57)	1.19 (0.89–1.60)	1.13 (0.75–1.70)	1.72 (1.08–2.74)*	1.08 (0.70–1.72)
High	1.55 (0.78–3.10)	1.08 (0.76–1.51)	2.07 (1.30–3.32)^a^	1.52 (0.89–2.59)	1.24 (0.75–2.06)
**Nationality**					
Belgian	2.57 (0.60–10.98)	0.76 (0.26–2.24)	0.28 (0.09–0.83)^a^	0.64 (0.29–1.42)	2.43 (0.95–6.25)
EU-countries	2.82 (0.52–15.35)	1.37 (0.43–4.32)	0.36 (0.10–1.29)	0.61 (0.18–2.05)	7.15 (1.68–30.51)^a^
Other countries	Ref.	Ref.	Ref.	Ref.	Ref.
**Income**					
Quintile 1	Ref.	Ref.	Ref.	Ref.	Ref.
Quintile 2	0.63 (0.31–1.27)	0.99 (0.70–1.39)	0.92 (0.55–1.55)	0.94 (0.54–1.63)	0.61 (0.36–1.06)
Quintile 3	1.29 (0.53–3.17)	0.99 (0.69–1.41)	1.17 (0.68–2.02)	1.05 (0.60–1.84)	0.6 (0.36–1.14)
Quintile 4	1.33 (0.64–2.77)	1.25 (0.86–1.81)	1.11 (0.61–2.03)	1.29 (0.69–2.40)	0.69 (0.37–1.28)
Quintile 5	1.05 (0.43–2.51)	1.16 (0.77–1.74)	1.34 (0.69–2.57)	0.70 (0.35–1.40)	1.37 (0.69–2.72)
**Region**					
Flanders	1.25 (0.74–2.09)	1.27 (1.02–1.59)^a^	1.64 (1.13–2.39)^a^	1.82 (1.20–2.75)^a^	2.50 (1.72–3.64)*
Brussels	1.70 (0.84–3.44)	1.06 (0.79–1.43)	1.29 (0.83–1.99)	1.03 (0.62–1.72)	2.30 (1.41–3.75)^a^
Wallonia	Ref.	Ref.	Ref.	Ref.	Ref.
**Perceived health**					
Good to very good	0.97 (0.57–1.65)	1.64 (1.26–2.14)^a^	1.76 (1.23–2.54)^a^	1.61 (1.10–2.36)^a^	1.10 (0.71–1.70)
Very bad to fair	Ref.	Ref.	Ref.	Ref.	Ref.
**Multimorbidity**					
Yes	Ref.	Ref.	Ref.	Ref.	Ref.
No	5.97 (3.06–11.67)^a^	0.47 (0.32–0.71)^a^	6.22 (3.86–10.03)*	15.40 (9.40–25.22)^a^	1.02 (0.61–1.71)
**Polypharmacy**					
Yes	Ref.	Ref.	Ref.	Ref.	Ref.
No	1.43 (0.77–2.68)	2.03 (1.42–2.90)^a^	1.26 (0.81–1.98)	0.70 (0.43–1.14)	2.59 (1.46–4.60)^a^

*CVDs* cardiovascular diseases (including hypertension), *COPD* chronic obstructive pulmonary disease; ^a^ Denotes significant difference between this group and the reference group. * adjusted for all other variables

## Discussion

In this study we assessed agreement between population-based administrative and survey data for ascertaining cases of diabetes, asthma, chronic obstructive pulmonary disease, cardiovascular diseases (including hypertension), Parkinson’s disease, thyroid disorders and epilepsy, for which BHIS data served as the gold standard. We also investigated the individual characteristics that could influence the agreement between both data sources.

Using the two data sources, we obtained inconsistent prevalence estimates in 3 out of the 7 CDs studied. Specifically, in CVDs (including hypertension), the prevalence was significantly higher in the BCHI data than in the BHIS data, while the inverse was true for COPD and asthma. The high prevalence of CVDs (including hypertension) according to the BCHI source (25%) compared to the BHIS prevalence (19%) could be explained by the use of drugs in this ATC group for other problems such as a high serum cholesterol for example. Some drugs may be assigned to two chronic diseases simultaneously, for example, beta-blockers are prescribed both for patients with hypertension and in patients with heart problems. As mentioned by Huber et al. in their study, an unique assignment of ATC-codes to heart diseases is challenging, and with the new trends in the use of various drugs for cardiac and hypertensive patients, a clear distinction between ATC-codes for cardiac diseases and hypertension is infeasible [9]. Therefore, we included hypertension in the BHIS based case definition of CVDs. The low prevalence of COPD and asthma in the administrative data could be explained by the fact that some people suffering from asthma or COPD do not necessarily take medications or less than 90 DDDs per year.

The estimated prevalence rate of diabetes mellitus from BCHI data is comparable to the one estimated in similar studies using health administrative database [9, 10, 22, 23], but higher than those in others comparable studies [5, 13]. Moreover, the prevalence of the respiratory illness (COPD, asthma) from BCHI is also comparable to those in similar in Netherlands, Italy and Swedish [5, 13, 24, 25]. Regarding the prevalence of Parkinson disease, thyroid disorders and Epilepsy, our results are in line with those reported by Francesco Chini et al. in Italy using a prescribed database [13] and by Huber et al. in Switzerland using medical and pharmacy claims data [9]. Considering the CVDs (including hypertension), our estimated prevalence was lower than the prevalence obtained by Huber et al. (29%) based on pharmacy data [9]. This difference could be explained by the CDs case definition used in their study: people were considered as having CD if they have at least one prescription in one of the generated ATC-groups CDs at the end of the reference year, while our definition was more selective (at least 90 DDDs per year which could correspond to several prescriptions (if small package) or more or less 3 months treatment per the given year.

We found that sensitivity of administrative CDs was good-to-fair for diabetes and CVDs and poor for the remaining CDs. Not surprisingly, the lowest sensitivity was for COPD and asthma. The sensitivity drop with the increase of the cut-off point of DDD, while the PPV increase.

CDs that are more prevalent or that are symptom-based may also be more reliably self-reported [26]. In our definition of CVDs in BHIS data source, we included hypertension, which may have contributed to increase the agreement between both data sources for CVDs.

The lower sensitivity of asthma (27.4%) in contrast with its relatively higher PPV (72.9%) in this study could be explained by the fact that most of the people suffering from a less severe case of asthma could not take up to 90 DDDs of the specific medication per year and those who reach that cut-off are certainly positive cases. Furthermore, in an exploratory analysis (results not shown), we found that 3 persons out of 10 suffering from this CD did not contact a health care professional in the past 12 months for that condition.

The agreement between the two data sources varies by participants’ sociodemographic characteristics and health status. However, this moderating effect varies in magnitude across CDs. Our results are consistent with findings in previous studies [3, 8, 27]. For instance, Lix et al. found that agreement between self-reported and medical records of chronic conditions was higher among younger age-groups and in the absence of comorbidity [3].

This study presents a number of strengths that deserve to be highlighted. First, the large sample size and the use of comprehensive administrative data, covering 99% of the Belgian population. It should be noted that not all countries have the opportunity to have such data. Thus, the existence of rich and detailed health insurance administrative data covering almost the entire population constitutes an added value for public health research in Belgium. Second, we calculated five agreement measures to enable comparison between data sources. Third, using individual record linkage, we further examined predictors that could affect the agreement between both data sources.

A number of limitations should also be acknowledged. One of the main limitations is that the case definition of CDs in the administrative data source was based on prescription drug codes dispensed in public pharmacies only and therefore drugs dispensed in the hospital settings were not included. Another limitation is the lack of additional information such as ICD-10 codes or other clinical diagnostic codes in the case ascertainment from administrative data source. Indeed, validation studies often include information from various sources in the algorithms: health surveys, ICD-10 codes, ATC codes, other clinical diagnostic codes, etc., and this provides much better measures of agreement [2, 3, 7, 10]. Finally, the BHIS data was used as the gold standard in this study because next to administrative data, it is the only source for obtaining population-based chronic disease prevalence estimates in Belgium. We acknowledged that self-reported data may not be an unbiased gold standard due to the risk of under-reporting or over-reporting of some chronic diseases. However, self-reported data have been used in previous studies to assess the validity of health administrative databases [20, 28, 29] and have shown higher agreement between these sources for chronic diseases that are more familiar to patients, well defined and require ongoing management [3, 20, 28, 30, 31]. Keeping this in mind, the CDs discussed in this study are sufficiently well known and defined that the risk of providing erroneous information from BHIS participants is negligible. Moreover, several studies have assessed the specificity of self-reported CDs compared to clinical diagnoses or medical records and have found that the specificity was at least 80% for asthma, hypertension, severe heart disease or heart attack, stroke, diabetes mellitus, epilepsy, and Parkinson’s disease [32].

## Conclusions

In conclusion, NHIDI’s algorithms are an acceptable alternative for the identification of cases of diabetes, cardiovascular diseases (without distinction of the different pathologies), Parkinson’s disease and thyroid disorders. On the basis of the current definition of CDs from BCHI data source, there is a significant underestimation of the number of patients suffering from asthma and COPD. Further research is needed to refine the definitions of CDs from administrative data by using other comparators (clinical data, data from general practitioners such as the Intego registry) or using different thresholds to enhance NIHDI algorithms.

## Supplementary Information

**Additional file 1 Table A1:** Characteristics of study population (*N* = 8474), HISLINK 2013, Belgium.

**Additional file 2 Table A2** Unadjusted Odds Ratios (95% CIs) for predictors of agreement between administrative and survey data for chronic diseases, HISLINK 2013, Belgium.

## Data Availability

The survey datasets and linked health administrative data analysed in the current study are not publicly available due to restrictions based in the General Data Protection Regulation (GDPR) on sensitive data such as personal health data. BHIS data contains sensitive and identifying information and therefore must only be made available upon request. Requests for data access may be made to the Social Security and Health Chamber of the Information Security Committee (hereinafter referred to as the “Social Security and Health Chamber”). Further information regarding the survey and the data access procedure can be found here: https://his.wiv-isp.be/nl/SitePages/ Procedure_gegevens2013.aspx.

## Abbreviations

ATC: Anatomical Therapeutic Chemical;
BCHI: Belgian compulsory health insurance
BHIS: Belgian health interview survey
CD: Chronic disease
COPD: Chronic obstructive pulmonary disease
CVD: Cardiovascular disease
DDD: Daily defined dose
HISLINK: Linkage between BHIS data and BCHI data
NIHDI: National Institute for Health and Disability Insurance
NPV: Negative predictive value
PPV: Positive predictive value
WHO: World Health Organization
