# Correction to: Comparing health insurance data and health interview survey data for ascertaining chronic disease prevalence in Belgium

**DOI:** 10.1186/s13690-020-00521-z

**Published:** 2020-12-21

**Authors:** Finaba Berete, Stefaan Demarest, Rana Charafeddine, Olivier Bruyère, Johan Van der Heyden

**Affiliations:** 1 SD Epidemiology and public health, Sciensano, Juliette Wytsmanstraat, 14 1050 Brussels, Belgium; 2 Department of Public Health, Epidemiology and Health Economics, University of Liège, Liège, Belgium; 3 WHO Collaborating Centre for Public Health aspects of musculoskeletal health and ageing, Department of Public Health, Epidemiology and Health Economics, University of Liege, Liège, Belgium

**Correction to: Arch Public Health 78, 120 (2020)**

**https://doi.org/10.1186/s13690-020-00500-4**

The original publication of this article [1] contained an error in the footnotes of Table 1 and an error in *the absolute difference for Diabetes mellitus* in Table 2 which were introduced during the publication process. The full tables are available via the original publication. In this correction article the incorrect and correct footnotes are shown. These errors do not alter the results and conclusion of the article. The original article has been updated.

**Table 1:**

**Incorrect:**

- ^a^ For people aged < = 50 years
- ^b^ For people aged > 50 years

**Correct:**

- ^a^ For people aged > 50 years
- ^b^ For people aged < = 50 years

**Table 2:**

**Incorrect**

**Table:** 

Chronic disease	Absolute difference^**b**^(E1-E2)
	% (95% CI)
Diabetes mellitus	−2.25 (−1.13 to 6.84)

**Correct**

**Table:** 

Chronic disease	Absolute difference^**b**^(E1-E2)
	% (95% CI)
Diabetes mellitus	−0.23 (−1.13 to 0.68).
