# Supplementary material for: Comparing health insurance data and health interview survey data for ascertaining chronic disease prevalence in Belgium
Source: Arch Public Health. 2020 Nov 17;78:120. doi: 10.1186/s13690-020-00500-4 (PMC7672883; doi:10.1186/s13690-020-00500-4)
Supplement: Supplementary file 1 — Additional file 1 Table A1: Characteristics of study population (N = 8474), HISLINK 2013, Belgium. [file 13690_2020_500_MOESM1_ESM.docx]

# Additional tables

Table A1 : Characteristics of study population (N= 8474), HISLINK 2013, Belgium

| **Characteristics** | **N** | **unweighted percentage  (sample)** | **weighted percentage**  **(population)** |
| --- | --- | --- | --- |
| **Gender** |  |  |  |
| Male | 4029 | 47.55 | 48.24 |
| Female | 4445 | 52.45 | 51.76 |
| **Age group** |  |  |  |
| 15-34 | 2323 | 27.41 | 28.75 |
| 35-54 | 2822 | 33.30 | 34.59 |
| 55-74 | 2377 | 28.05 | 25.90 |
| 75+ | 952 | 11.23 | 10.76 |
| **Education** |  |  |  |
| Low | 2193 | 25.88 | 23.58 |
| Intermediate | 2719 | 32.09 | 33.93 |
| High | 3443 | 40.63 | 41.57 |
| Missing | 119 | 1.40 | 0.92 |
| **Nationality** |  |  |  |
| Belgian | 7461 | 88.05 | 91.08 |
| EU-countries | 603 | 7.12 | 5.01 |
| Other countries | 404 | 4.77 | 3.85 |
| Missing | 6 | 0.07 | 0.06 |
| **Income** |  |  |  |
| Quintile 1 | 1575 | 18.59 | 15.72 |
| Quintile 2 | 1298 | 15.32 | 15.22 |
| Quintile 3 | 1493 | 17.62 | 18.75 |
| Quintile 4 | 1484 | 17.51 | 19.97 |
| Quintile 5 | 1571 | 18.54 | 20.57 |
| Missing | 1053 | 12.43 | 9.77 |
| **Region** |  |  |  |
| Flanders | 2927 | 34.54 | 58.11 |
| Brussels | 2241 | 26.45 | 10.09 |
| Wallonia | 3306 | 39.01 | 31.80 |
| **Perceived health** |  |  |  |
| Good to very good | 4719 | 55.69 | 61.13 |
| Very bad to fair | 1467 | 17.31 | 17.27 |
| Missing | 2288 | 27.00 | 21.60 |
| **Multimorbidity** |  |  |  |
| Yes | 1233 | 14.55 | 13.70 |
| No | 7234 | 85.37 | 86.27 |
| Missing | 7 | 0.08 | 0.03 |
| **Polypharmacy** |  |  |  |
| Yes | 902 | 10.64 | 9.67 |
| No | 7563 | 89.25 | 90.26 |
| Missing | 9 | 0.11 | 0.06 |
