# Supplementary material for: Comparing health insurance data and health interview survey data for ascertaining chronic disease prevalence in Belgium
Source: Arch Public Health. 2020 Nov 17;78:120. doi: 10.1186/s13690-020-00500-4 (PMC7672883; doi:10.1186/s13690-020-00500-4)
Supplement: Supplementary file 2 — Additional file 2 Table A2 Unadjusted Odds Ratios (95% CIs) for predictors of agreement between administrative and survey data for chronic diseases, HISLINK 2013, Belgium. [file 13690_2020_500_MOESM2_ESM.docx]

Table A2 Unadjusted odds Ratios (95% CIs) for predictors of agreement between administrative and survey data for chronic diseases, HISLINK 2013, Belgium

|  | **Diabetes** | **CVDs** | **COPD** | **Asthma** | **Thyroid**  **disorders** |
| --- | --- | --- | --- | --- | --- |
| **Gender** |  |  |  |  |  |
| Male | Ref. | Ref. | Ref. | Ref. | Ref. |
| Female | 0.9 (0.6-1.4) | 0.8 (0.7-1.0) | 0.7 (0.6-0.9) | 0.8 (0.5-0.9) | 1.2 (0.7-2.0) |
| **Age group** |  |  |  |  |  |
| 15-34 | 14.6 (6.6-32.0) | 10.4 (7.3-14.7) | 5.0 (2.8-9.0) | 1.0 (0.7-1.5) | 2.2 (1.0-4.7) |
| 35-54 | 2.0 (1.2-3.3) | 2.2 (1.7-2.7) | 1.9 (1.4-2.7) | 1.4 (0.9-2.0) | 1.6 (0.9-2.2) |
| 55-74 | Ref. | Ref. | Ref. | Ref. | Ref. |
| 75+ | 0.8 (0.5-1.3) | 0.5 (0.4-0.6) | 0.7 (0.5-1.0) | 1.4 (0.9-2.3) | 1.2 (0.5-2.7) |
| **Education** |  |  |  |  |  |
| Low | Ref. | Ref. | Ref. | Ref. | Ref. |
| Intermediate | 1.6 (1.0-2.7) | 2.0 (1.6-2.5) | 1.7 (1.3-2.5) | 1.8 (1.2-2.6) | 1.3 (0.7-2.5) |
| High | 3.5 (2.2-5.6) | 2.5 (2.0-3.1) | 1.8 (1.3-2.6) | 1.8 (1.3-2.6) | 2.2 (1.2-4.2) |
| **Nationality** |  |  |  |  |  |
| Belgian | 1.3 (0.5-3.8) | 0.6 (0.3-1.0) | 0.3 (0.1-0.8) | 0.8 (0.4-1.1) | 0.2 (0.1-1.0) |
| EU-countries | 1.0 (0.3-3.8) | 1.0 (0.5-1.8) | 0.2 (0.1-0.7) | 0.7 (0.3-1.8) | 0.3 (0.1-1.7) |
| Other countries | Ref. | Ref. | Ref. | Ref. | Ref. |
| **Income** |  |  |  |  |  |
| Quintile 1 | Ref. | Ref. | Ref. | Ref. | Ref. |
| Quintile 2 | 0.6 (0.3-1.1) | 0.7 (0.6-1.0) | 0.8 (0.6-1.3) | 1.3 (0.8-1.9) | 0.9 (0.4-2.3) |
| Quintile 3 | 1.3 (0.6-2.7) | 1.0 (0.8-1.3) | 1.3 (0.9-2.0) | 1.7 (1.1-2.7) | 1.0 (0.5-2.2) |
| Quintile 4 | 1.6 (0.8-3.1) | 1.4 (1.0-1.9) | 1.9 (1.2-3.1) | 1.9 (1.2-3.0) | 1.4 (0.6-3.3) |
| Quintile 5 | 1.8 (0.9-3.4) | 1.4 (1.1-1.9) | 2.9 (1.8-4.7) | 1.3 (0.8-2.2) | 1.4 (0.7-3.1) |
| **Region** |  |  |  |  |  |
| Flanders | 1.3 (0.9-2.0) | 1.2 (1.0-1.4) | 1.6 (1.2 -2.1) | 2.0 (1.5-2.9) | 1.4 (0.8-2.4) |
| Brussels | 1.5 (1.0-2.3) | 1.3 (1.0-1.6) | 1.6 (1.1-2.2) | 0.9 (0.6-1.3) | 1.9 (1.0-3.5) |
| Wallonia | Ref. | Ref. | Ref. | Ref. | Ref. |
| **Perceived health** |  |  |  |  |  |
| Good to very good | 2.6 (1.5-4.3) | 2.5 (2.1-3.1) | 4.3 (3.1-5.8) | 2.8 (2.0-4.0) | 6.2 (3.3-11.6) |
| Very bad to fair | Ref. | Ref. | Ref. | Ref. | Ref. |
| **Multimorbidity** |  |  |  |  |  |
| Yes | Ref. | Ref. | Ref. | Ref. | Ref. |
| No | 7.1 (4.6-10.9) | 1.7 (1.4-2.1) | 10.9 (8.2-14.5) | 7.4 (5.4-10.0) | 2.2 (1.3-3.7) |
| **Polypharmacy** |  |  |  |  |  |
| Yes | Ref. | Ref. | Ref. | Ref. | Ref. |
| No | 4.8 (3.0-7.5) | 3.8 (3.1-4.7) | 5.2 (3.9-6.9) | 2.2 (1.6-3.1) | 3.6 (2.1-6.0) |

*CVDs = cardiovascular diseases (including hypertension) ; COPD = chronic obstructive pulmonary disease.*
